# Extragalactic background light measurements and applications

**DOI:** 10.1098/rsos.150555

**Published:** 2016-03-09

**Authors:** Asantha Cooray

**Affiliations:** Department of Physics and Astronomy, University of California, Irvine, CA 92697, USA

**Keywords:** cosmology, galaxies: galaxy evolution

## Abstract

This review covers the measurements related to the extragalactic background light intensity from γ-rays to radio in the electromagnetic spectrum over 20 decades in wavelength. The cosmic microwave background (CMB) remains the best measured spectrum with an accuracy better than 1%. The measurements related to the cosmic optical background (COB), centred at 1 μm, are impacted by the large zodiacal light associated with interplanetary dust in the inner Solar System. The best measurements of COB come from an indirect technique involving γ-ray spectra of bright blazars with an absorption feature resulting from pair-production off of COB photons. The cosmic infrared background (CIB) peaking at around 100 μm established an energetically important background with an intensity comparable to the optical background. This discovery paved the way for large aperture far-infrared and sub-millimetre observations resulting in the discovery of dusty, starbursting galaxies. Their role in galaxy formation and evolution remains an active area of research in modern-day astrophysics. The extreme UV (EUV) background remains mostly unexplored and will be a challenge to measure due to the high Galactic background and absorption of extragalactic photons by the intergalactic medium at these EUV/soft X-ray energies. We also summarize our understanding of the spatial anisotropies and angular power spectra of intensity fluctuations. We motivate a precise direct measurement of the COB between 0.1 and 5 μm using a small aperture telescope observing either from the outer Solar System, at distances of 5 AU or more, or out of the ecliptic plane. Other future applications include improving our understanding of the background at TeV energies and spectral distortions of CMB and CIB.

## 1. Introduction

The extragalactic background light (EBL) is the integrated intensity of all of the light emitted throughout the history of the universe across the whole of the electromagnetic spectrum. While EBL is sometimes defined as the extragalactic intensity spectrum from UV to infrared (e.g. see review in Dwek & Krennrich [1]), the total energy content of the universe in the electromagnetic spectrum spans close to 20 decades in wavelength from γ-rays to radio. Across this whole range, the EBL spectrum captures cosmological backgrounds associated with either primordial phenomena, such as the cosmic microwave background (CMB), or photons emitted by stars, galaxies and active galactic nuclei (AGN) due to nucleosynthesis or other radiative processes, including dust scattering, absorption and reradiation. The EBL may also contain signals that are diffuse and extended, including high-energy photons associated with dark matter particle decays or annihilation.

In the UV to infrared portion of the electromagnetic spectrum, the EBL spectrum captures the redshifted energy released from all stars and galaxies throughout cosmic history, including first stellar objects, primordial black holes and proto-galaxies. If precisely measured, the EBL spectrum can be used as a constraint on models of galaxy formation and evolution, while providing an anchor that connects global radiation energy density to star formation, metal production and gas consumption. The microwave background spectrum at micrometre to millimetre radio wavelengths, associated with CMB photons, has been measured to a precision better than 1% and is described by a blackbody spectrum with a temperature of 2.7260±0.0013 K [2]. Such a measurement is facilitated by the fact that the CMB is the brightest of the EBL components with a factor of 30–40 higher energy density than the next brightest background at optical to infrared wavelengths. The CMB is also a well-known probe of cosmology. The anisotropies come from both primordial physics, at the epoch of last scattering when electrons and protons first combined to form hydrogen, and secondary effects during the propagation of photons. The latter includes effects associated with both gravitational and scattering effects. The angular power spectrum of CMB spatial anisotropies has now been measured down to a few arcminute scales with Planck and has been used to determine cosmological parameters such as the energy density contents, the spatial curvature, spectral index of primordial density perturbations laid out after an inflation epoch, among others.

Despite the limitations on the accuracy of existing EBL intensity measurements there have been some key breakthroughs due to intensity measurements of the sky. A classic example is the cosmic IR background (CIB) peaking at 100 μm. Its intensity was measured with instruments such as DIRBE [3] and at wavelengths beginning 250 μm with FIRAS [4], both on COBE. The EBL intensity peaking at 100 μm was found to be roughly comparable to that of the optical background, suggesting that CIB at long wavelengths is energetically as important as the optical/near-IR background dominated by galaxies. This then motivated high-resolution far-infrared and sub-millimetre imaging from both space and ground and with increasing aperture sizes and sensitivity more of the CIB has been resolved to point sources. These point sources are mainly dusty, star-forming galaxy (SFG) and starbursting galaxy at high redshifts (see review in Casey *et al.* [5]). Their role in galaxy formation and galaxy evolution remains one of the key topics in sub-millimetre astronomical observations today.

With a precise measurement of the EBL intensity spectrum, a cosmic consistency test can be performed as a function of the wavelength by comparing the integrated light from all galaxies, stars, AGN and other point sources, to the EBL intensity. Any discrepancies suggest the presence of new, diffuse emission that is unresolved by telescopes. The possibilities for new discoveries with profound implications for astronomy range from recombination signatures during reionization and diffuse photons associated with dark matter annihilation and their products. Related to the last scientific possibility important studies have been carried out, with more expected over the coming years, as to whether there is a diffuse signature at GeV energy scales in the cosmic γ-ray background (CGB) as measured by the Fermi-LAT that can be ascribed to dark matter (e.g. [6]).

In addition to the total EBL intensity significant information on the sources of emission and their nature comes from measurements that focus on the anisotropies of the intensity across the sky. These are in general quantified and measured in terms of the angular power spectrum or the correlation function. The well-studied example in the literature is the anisotropy power spectrum of the CMB, resulting in high-precision cosmological parameter measurements (e.g. [7]). Anisotropy power spectra have also been measured for CIB, cosmic optical background (COB) and CGB leading to inferences on the properties of the source populations present at these wavelengths, especially on certain physical details related to the faint sources that are below the individual point source detection level.

The existing EBL intensity measurements are due to a combination of ground and space-based observations of the sky. Direct absolute intensity measurements must account for a variety of foregrounds both within the Solar System, such as zodiacal light at optical and infrared wavelengths, and in the Milky Way, such as the Galactic emission at radio, infrared, X-ray or γ-ray wavelengths. A good example of an indirect technique to measure EBL is the use of absorbed TeV spectra of individual blazars and other AGNs at cosmological distances to infer the number density of intervening infrared photons that are responsible for electron–positron pair production by interactions with TeV photons. This has led to the best determined COB measurements in the literature, especially given the fact that modelling and removing zodiacal light remains a challenge for direct EBL intensity measurements around 1 μm.

**Figure 1.**
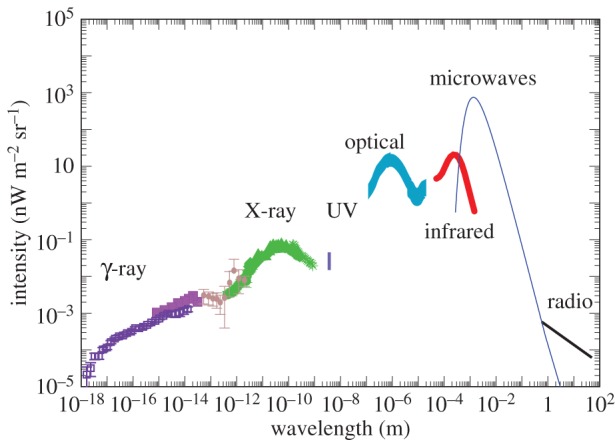
Intensity of the extragalactic background (*νI*_*ν*_ in units of nW m^−2^ sr^−1^) as a function of the wavelength in metres. We combine the existing measurements from the literature to highlight the best determined estimates for the background from γ-ray to radio. The CMB has the least uncertainty as the spectrum is determined to better than 1%. COB has large uncertainties involving direct measurements due to uncertain removal of the zodiacal light foreground. Here we show the indirect estimate of EBL at optical wavelengths based on the TeV/γ-ray absorption spectra of distant blazars. The UV/soft X-ray background at a wavelength of 10–100 nm remains unexplored. From left to right in increasing wavelength, the plotted datasets are: Fermi-LAT (the total extragalactic background composed of diffuse and resolved point sources) [8] and EGRET [9] (we have removed three data points from Strong *et al.* [10] at highest energies) in the γ-ray spectrum, COMPTEL (filled circles) [11] between γ- and X-rays, HEAO1 A2 and A4 [12,13], INTEGRAL [14], SWIFT/BAT [15], Nagoya balloon experiment [16], SMM [17], ASCA [18] and RXTE [19] in the hard to soft X-ray regime in green symbols, DXS and CHIPS in soft X-rays/extreme UV (as discussed in Smith *et al.* [20] as a line at 0.25 keV), HESS in optical [21] (see figure 2 for other measurements), DIRBE [3] and FIRAS [22] in the far-infrared, FIRAS at microwaves [23,24] and ARCADE [25] in the radio. The area under each of these backgrounds captures the total energy density of the photons in each of those wavelength regimes. From γ-rays to radio the integrated intensity values in units of nW m^−2^ sr^−1^ for key EBL components are approximately 0.015 (γ-ray), approximately 0.3 (X-ray), 0.01–0.02 (lower and upper limits at 4.9 nm for extreme UV), 24±4 (with an additional ±5 systematic; optical), approximately 30±10 (CIB), 960 (CMB) and less than 0.001 (radio).

We summarize existing EBL intensity measurements in figure 1 where we plot the spectral intensity λ*I*_λ_ as a function of the wavelength λ. In this figure, the area under each of the spectral components represents the total energy density associated with each of those backgrounds. Those values are listed in the caption of figure 1 where the estimates were made using a statistical average of existing results from the literature. In most of these measurements large systematics, associated with foreground models, are likely to be still present. Here we briefly outline the techniques, foregrounds and systematics associated with EBL measurements. We also discuss their applications for astrophysical and cosmological studies and briefly summarize studies related to spatial anisotropies. We cover from short to long wavelengths starting from the γ-ray background.

### 1.1 γ-ray

The early measurements of the CGB intensity came from SAS-2 between 40 and 300 MeV in 1978 [49], followed by EGRET between 40 MeV and 10 GeV in 1998 [9,10]. These measurements have been superseded in this decade by Fermi-LAT covering 100 MeV to 800 GeV with roughly 25–30 times better sensitivity than EGRET, as well as overall an improvement in the flux calibration. The CGB spectrum measured by Fermi-LAT shows a cutoff at energy scales around 280 GeV [8]. Below this cutoff, the spectrum can be described by a single power law with a spectral index about 2.3 (±0.05). The cutoff is explained as the disappearance of the high-energy photons that are pair-producing via interactions with the infrared background photons that we discuss later [50–53].

The CGB spectrum below the cutoff is mostly explained in terms of a combination of AGNs in the form of blazars and γ-ray emission from SFGs. Small, but non-negligible depending on the exact energy, comes from millisecond pulsars, Type Ia supernovae and γ-rays from galaxy clusters. At energies above approximately 50 GeV blazars are able to fully account for the background, with an estimate of $86_{-14}^{+16}$% explained by extrapolated blazar counts in Fermi-LAT Collaboration [54]. Between 0.1 and 50 GeV, blazars account for about 20% of the CGB. The rest are due to other populations of AGNs and SFGs. We refer the reader to a comprehensive review by Fornasa & Sánchez-Conde [55] on the source populations contributing to CGB and existing population models in the literature.

The literature also considers the possibility of a dark matter-induced signal in the γ-ray background (see [56] for a review). This could be from dark matter that decays into standard particles (e.g. [57,58]) or due to annihilation products [59] (see also [60]). These γ-rays will form an anisotropic signal associated with our Galaxy and mostly an isotropic background that diffusely traces the dark matter halos of all other galaxies. A direct detection of a signal associated with dark matter decay has been attempted towards the Galactic centre (e.g. [61]) and nearby dwarf galaxies that are considered to be dark matter rich (e.g. [62,63]). The claims of γ-ray excesses towards the Galactic centre have been questioned as whether due to pulsars or other astrophysical foregrounds (e.g. [64,65]), while the signal towards dwarf galaxies remains at the level of a 3*σ* detection (e.g. [66]).

Owing to spatial resolution and the all-sky nature of the CGB measurements, Fermi-LAT also provides a treasure trove of data beyond the energy spectrum. In particular, anisotropies or spatial fluctuations of the CGB have been pursued to study the nature of faint sources that can account for the small diffuse signals in the CGB below the point source detection of current high-energy instruments. The angular power spectrum of the CGB is mostly Poisson or shot-noise like between multipole ell ranges of 150–500, corresponding to 30 arcmin to 3° angular scales on the sky (e.g. [67]). Moving beyond auto spectra, cross-correlations of the anisotropies can also be pursued (e.g. [68]). For an example, the diffuse CGB signal from all of decaying dark matter in the universe can be studied via cross-correlations with large-scale structure tracers of the same dark matter, such as weak lensing maps (e.g. [69]). Existing cross-correlation attempts using Fermi-LAT data include both the CMB lensing map from Planck [70] and galaxy weak lensing in the CFHT Lensing Survey [71]. Such studies will be improved in the future with wider area galaxy lensing surveys such as those expected from the dark energy survey and large synoptic survey telescope.

Over the next decade, CGB studies will be extended to higher energies with the Cherenkov Telescope Array (CTA) that is expected to cover 10 GeV to 10 TeV energy range [72]. CTA will allow studies related to CGB fluctuations at higher energies, especially TeV scales where there are still no reliable measurements on the spectrum or intensity fluctuations. We also lack a complete understanding of the sources that contribute to CGB at 1–10 MeV scales, below the 100 MeV sensitivity of Fermi-LAT. This background spectrum based on EGRET and COMPTEL (figure 1) suggests the possibility of a smooth connection to the cosmic X-ray background (CXB) at 10–100 keV energy scales, though subject to large uncertainties in COMPTEL measurements. If continue to be confirmed such a smooth transition to X-rays suggests a different source population for the MeV background than the background GeV energies and the leading possibility is a combination of Seyfert galaxies and flat-spectrum radio quasars that appear as bright MeV sources. Owing to large uncertainties with EGRET and COMPTEL background measurements at these energy scales, however, we recommend a future experiment to improve measurements at MeV energy scales.

### 1.2 X-ray

CXB is generally divided into hard and soft energies around an energy scale of 2 keV. Early measurements of the hard CXB intensity came from HEAO1 between 2–30 keV and 10–400 keV with A2 and A4 instruments. These measurements showed that the spectrum can be described by: $I(E)=7.9\times 10^{-0.29}\exp[-E/41.1\;\mathrm{keV}]\;\mathrm{keV}\;cm^{-2}\;s^{-1}\;sr^{-1}\;\mathrm{ke}V^{-1}$, consistent with thermal bremsstrahlung radiation with a temperature approximately 40 keV (e.g. [12,13,73]). Bulk of the energy density of CXB is thus at 30 keV, but understanding the sources at this energy has been slow. Previous surveys with SWIFT/BAT and Integral [14,15] only resolved 1% of the background to point sources at 30 keV.

Recent models show that in order to match both the redshift distribution of the faint X-ray sources in deep images with the Chandra X-ray Observatory and the overall CXB spectrum requires an evolving ratio of Type 1 (AGNs with visible nuclei) to Type 2 (with obscured broad-line regions) sources, such that there are more Type 2 Seyferts at higher redshifts (e.g. [74–76]). A significant improvement on the fraction resolved to point sources at more than 10 keV is expected with NuStar that is now taking data [77]. The simple expectation is that deep surveys of NuStar should resolve more than 30% of the background at approximately 20 keV, involving mainly highly absorbed AGNs [78]. There are some indications for the presence of such absorbed AGNs as IR-bright sources in AKARI, with roughly 50% of the sample currently undetected in deep Chandra surveys (e.g. [79]).

The soft CXB has been measured with ROSAT down to 0.1 keV energies. At such low energies, intensity measurements start to become challenging due to the Galactic signal, and there is a clear indication for thermal emission from hot gas with a temperature of 10^6^ K associated with a hot component of the interstellar medium or the Galactic halo. Deep ROSAT imaging data resolved 80% of the CXB at soft X-ray energies of 1 keV to discrete sources, mainly bright AGNs. Chandra X-ray Observatory, with spatial resolution at the level of 0.5 arcsec, and XMM-Newton have resolved more than 90% of the X-ray background at energies between 0.5 and 2 keV and more than 80% at hard 2–9 keV energies [78,80]. The dominant source is AGNs with a non-negligible contribution from galaxy clusters (e.g. [81]) and starbursting galaxies (e.g. [82]). The uncertainty in the unresolved intensity is not in the source population but in the overall normalization of the total X-ray background intensity.

With the background resolved to individual sources, anisotropy measurements of the CXB intensity and their applications have been limited when compared with similar studies at other wavelengths. In principle, anisotropy studies can uncover diffuse X-ray sources or faint sources below the detection level. A recent example is the use of Chandra deep images to study the X-ray background fluctuations in combination with fluctuations measured with Spitzer at 3.6 μm [83]. This signal has been explained as due to the infrared and X-ray emission from direct collapse black holes (DCBHs) during reionization [84]. An X-ray surveyor, such as the planned Athena mission, should facilitate more detailed studies on the diffuse background, unresolved sources such as DCBHs, and anisotropy and cross-correlation studies.

### 1.3 Ultraviolet

EBL measurements at UV wavelengths exist with GALEX at 150 nm [85], with Voyager 1 and 2 at 110 nm [86], and with Voyager UVS at 100 nm [34], though subject to both large statistical and systematic uncertainties. In the extreme UV (EUV) wavelengths below 100 nm, and down to 10 nm at energy scales of 0.1 keV in soft X-rays, there are no useful measurements of the cosmic UV background in the literature (figure 1). While technological developments can be expected, a measurement of the extragalactic EUV background will likely remain challenging due to absorption of the extragalactic photons by neutral hydrogen in our Galaxy and the intergalactic medium at wavelengths below 91.2 nm. Furthermore, the Galactic soft X-ray/EUV background presents a considerable foreground that limits a reliable measurement of the UV background. And the next best measurements are in X-rays from a wavelength of 5 nm corresponding to energies of 0.25 keV.

While a reliable background intensity might be challenging, further EUV observations are warranted since it is understood that bulk of the baryons exists in the warm intergalactic medium [87] with signatures that involve emission and absorption lines around 50 nm [88]. Based on Extreme UltraViolet Explorer data, there are some indications that certain galaxy clusters like Coma show excess EUV emission, especially at energies between 65 and 200 eV [89]. While the wavelength regime between 10 and 100 nm remains crucial for further exploration, we recommend further attempts between 100 and 1000 nm in the UV as there are possibilities for some significant discoveries on the nature of warm intergalactic medium. Instruments on the planetary spacecraft to the outer Solar System may continue to provide opportunities for UV background measurements, similar to past attempts with Voyager [34,86]. In this respect, one near-term possibility would be the use of New Horizons’ Alice UV spectrometer [90] for a new background measurement at wavelengths around 140–180 nm.

### 1.4 Optical/near-infrared

At optical and near-IR wavelengths between 0.1 and 5 μm, the EBL intensity is predominantly due to stellar emission from nucleosynthesis throughout cosmic history (see Hauser & Dwek [47] for a review). The COB spectrum also includes radiative information from the reionization epoch. Owing to redshifting of the UV photons to near-infrared emission from primordial sources is primarily at wavelengths longward of 1 μm [91,92]. This includes diffuse Ly-*α* and free–free radiation in addition to direct emission by stars and mini-quasars (e.g. [93,94]).

At optical and near-IR, the few attempts at absolute measurements involve DIRBE on COBE in several band-passes between 1.25 and 240 μm [3,26–30], IRTS, a small JAXA mission, between 1 and 4 μm [31], Voyager [34] and Hubble [33,35,95]. Because DIRBE’s confusion limit was 5th magnitude at 2.2 μm, all recent EBL measurements using DIRBE require subtraction of stellar light using ancillary measurements, such as 2MASS [27,30]. While Hubble has been used for optical [33,95] and far-UV [35] EBL measurements, the instrument was not designed for absolute photometry and required a careful subtraction of instrumental emission and baselines (e.g. dark current). Those measurements are subject to large uncertainties (e.g. [96]).

The dominant limitation for direct EBL intensity spectrum at these wavelengths is the zodiacal light associated with scattered solar light from micrometre-size interplanetary dust (IPD) particles near the Earth’s orbit. Existing measurements with DIRBE on COBE make use of a model to remove zodiacal light [97] or slight variations [27]. The zodiacal light foreground limits the accuracy of EBL intensity to roughly an order of magnitude at wavelengths about 1 μm [3,27–29,31,32]. Techniques to remove zodiacal light include monitoring of Fraunhofer lines in the dust scattered spectrum relative to the solar spectrum and use of the equivalent width of the lines to estimate the column density of dust. In the case of Hubble results on the optical background, the zodiacal emission based on the observed strength of the reflected Fraunhofer lines from a ground-based measurement [95]. The sounding rocket experiment CIBER [98] is capable of absolute photometry [99] and results related to the optical/near-IR EBL are soon expected. If Spitzer/IRAC shutter operations are allowed and successful, a carefully planned programme should also be able to improve the absolute EBL measurements at 3.6 and 4.5 μm over the coming years.

**Figure 2.**
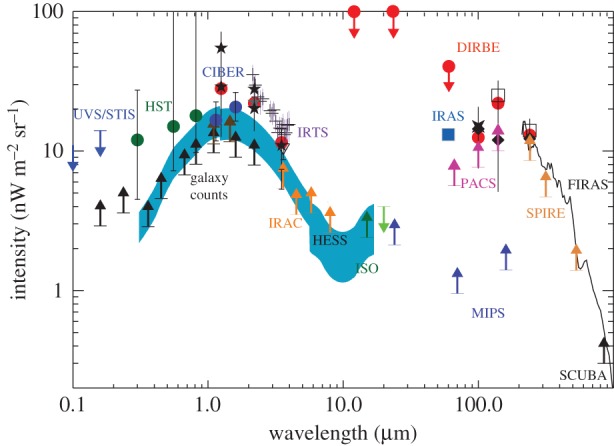
The cosmic optical and infrared background light from 0.1 to 100 μm. The data points with error bars are direct estimates using DIRBE (red circles: [26,27]; stars: [28] at 1.25 and 2.2 μm; [29] at 2.2 and 3.5 μm; [30] at 2.2 and 3.5 μm; open squares at 140 and 240 μm; [3]), IRTS (purple crosses; [31]), Spitzer at 3.6 μm (open triangle; [32]), Hubble (green circles; [33]), UVS/STIS (blue upper limits; [34,35]), CIBER (blue circles; model-dependent based on fluctuation measurements; [36]), FIRAS (black line; with an overall uncertainty of 30% between 200 μm and 1.2 mm; [22] also [4]) and IRAS (blue square; 60 μm fluctuation-based estimate of EBL with IRAS; [37]). The lower limits to the EBL are from integrated or source counts using Hubble [38,39], Spitzer/IRAC [40], ISO [41], Spitzer/MIPS [42,43], Herschel/PACS [44], Herschel/SPIRE [45] and SCUBA [46]. The blue shaded region is the estimate of EBL using the HESS TeV blazar absorption spectra [21]. Apart from recent Herschel measurements, CIBER and the estimate of EBL from HESS, all other measurements plotted here are tabulated in Hauser & Dwek [47]. This figure is based on a previous figure by Dole *et al.* [48] that summarized these EBL and integrated galaxy count measurements.

In figure 2, we summarize EBL intensity measurements between optical and IR wavelengths using absolute photometry, model-dependent estimates based on the EBL fluctuations, and the integrated galaxy light (IGL) from galaxy counts (lower limits). In general, the summed contribution of galaxies to the EBL does not reproduce the EBL measured by absolute photometric instruments. For example, at λ=3.5 μm the EBL measured by DIRBE from absolute photometry is 13.0±4.8 nW m^−2^ sr^−1^ [30], while the deepest pencil beam surveys with Spitzer at 3.6 μm give 6−9 nW m^−2^ sr^−1^ [40,100], with the best determination of $9.0_{-0.9}^{+1.7}\;$nW m^−2^ sr^−1^ [32]. At shorter wavelengths centred around 1 μm, and especially considering EBL measurements from IRTS [31], this divergence is even more pronounced.

Note that model-dependent fluctuations-based estimates of EBL are in between IGL and absolute photometry measurements (at 1 μm, such estimates of EBL are from CIBER [36]; figure 2). Fluctuation measurements and angular power spectra of source-subtracted optical and near-IR background intensity have been presented with Spitzer at 3.6 μm and above [101–103], AKARI at 2.4, 3.2 and 4.1 μm [104], Hubble/NICMOS at 1.1 and 1.6 μm [105], CIBER at 1.1 and 1.6 μm [36], and Hubble/ACS and WFC3 in five bands from 0.6 to 1.6 μm [106]. These measurements generally reveal a picture in which source counts, mainly galaxies, dominate the fluctuations with some evidence for additional components such as intra-halo light (IHL) [36,107], associated with diffuse stars in extended dark matter halos due to galaxy mergers and tidal stripping, and a signal from galaxies present during reionization at *z*>8 [106]. Spitzer fluctuations have been also cross-correlated with far-infrared maps from Herschel/SPIRE [108] and X-ray from Chandra [83]. The former allows a quantification of the total dust content as a function of redshift while the latter has been used to argue for the presence of primordial DCBHs [84]. Over the coming decade significant improvements in the study of near-IR and optical fluctuations will come from planned cosmological missions such as Euclid and WFIRST. The small explorer SPHEREx [109], recently selected by NASA for a Phase A study, has the ability to conduct three-dimensional intensity mapping of spectral lines such as H*α* at *z*∼2 and Ly-*α* at *z*>6 during reionization over large areas on the sky.

A leading possibility for the large difference between absolute photometry EBL and IGL is likely an unsubtracted foreground component, such as an underestimate of the zodiacal light signal [110]. If this difference, however, is real it would have significant implications given that the nature of the emission source must be diffuse and not point-like similar to galaxies. Fortunately, there is also a third technique to measure the EBL. Given the limitation of direct measurements possibly due to foregrounds, currently the best estimates of optical/near-IR EBL come from this indirect technique that makes use of the absorbed TeV/GeV spectra to constrain the optical and infrared background due to pair production (shaded region in figure 2 from Abramowski *et al.* [21]). The modelling requires an intrinsic spectrum for each blazar, but since this is not observed or available, EBL is inferred through statistical techniques that make use of a large sample of blazars over a wide range of cosmological distances. The existing measurements come from High Energy Stereoscopic System (HESS) array in Namibia [21,51] and Fermi-LAT [50,111]. The discrepancy between absolute photometry and IGL-implied intensity is less severe when comparing galaxy counts to the EBL inferred from absorbed TeV spectra. The measurements are such that deep galaxy counts have effectively resolved all of the optical and near-IR photons to individual galaxies. The overall uncertainties, however, are still that the measurements leave the possibility for small signals such as IHL and reionization consistent with fluctuation measurements.

**Figure 3.**
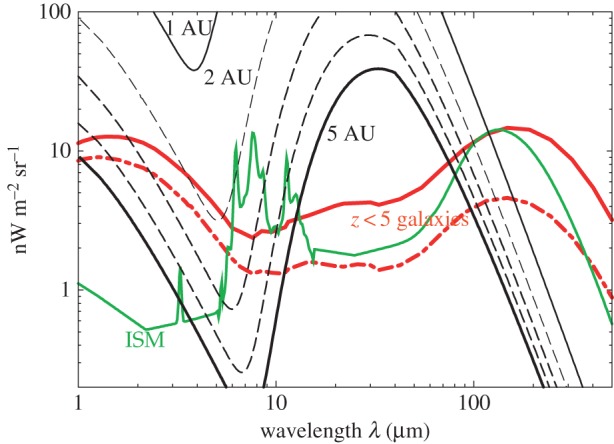
Zodiacal light intensity as a function of the radial distance from the Sun, based on the Pioneer 10 dust density estimates [112] normalized to a radial profile from the Sun of *r*^−1.5^. For reference, we also show the optical to near-IR spectrum of the Galactic interstellar medium (ISM) and two predictions from semi-analytical models on the total IGL from *z*<5 galaxy populations (adapted from Primack *et al.* [113]). The zodical light intensity estimates as a function of the radial distance are based on the calculations for ZEBRA concept instrument for the outer Solar System in Cooray *et al.* [114].

Given the large uncertainties, including systematics, with TeV measurements, and especially absolute photometry measurements, it is crucial that we improve on the optical and near-IR EBL intensity level. It is also clear that simply repeating an absolute photometry experiment like DIRBE or IRTS at 1 AU will not improve the current EBL spectrum at wavelengths less than 5 μm due to limitations coming from the foreground model. Improvements in EBL measurements will only come with a parallel improvement in our understanding of the IPD distribution in the Solar System, if measurements are limited to 1 AU, or from observations that are conducted outside of the zodiacal light dust cloud. In figure 3, we show the expected zodiacal light intensity as a function of the radial distance from the Sun, based on the *in situ* dust measurements from Pioneer 10; the dust density is dropping more rapidly than the *r*^−1^ profile expected from the Poynting–Robertson effect [112]. At distances of Jupiter, these dust density measurements suggest a decrease in the zodiacal light intensity of roughly two orders of magnitude from the intensity level near Earth orbit. One possibility is the out-of-Zodi EBL measurements, such as the proposed piggy-back ZEBRA instrument on a planetary spacecraft to Jupiter or Saturn distances, or to travel outside of the ecliptic plane. Given the relatively small cost of the instrument necessary for the required observations we encourage attempts to measure EBL as a by-product of planetary spacecraft that explore the outer Solar System [114].

### 1.5 Far-infrared

Absolute photometry measurements between 10 and 1000 μm, corresponding to the far-infrared EBL peak at around 100 μm (figure 2), are mainly from DIRBE [3] and FIRAS [4,22]. In general, these measurements have an overall uncertainty at the level of 30%, all due to uncertain corrections associated with the foreground sky model. This background is generally referred to as the CIB in some literature, especially in the context of CMB experiments that also overlap in frequency ranges as the sub-millimetre wavelengths.

With the absolute photometry measurements establishing a cosmologically important energy density in the universe at long infrared wavelengths, subsequent observations have focused on resolving this background to point sources with large aperture telescopes. While the fraction resolved was low with Spitzer/MIPS at 70 and 160 μm, significant improvements in our ability to detect and study distant galaxies came over the last 5 years with Herschel/PACS and SPIRE between 70 and 500 μm. In particular, Herschel/SPIRE resolved 15% (250 μm) to 5% (500 μm) of the background directly to sources [115]. Using statistics such as *P*(*D*), probability of deflections, in deep SPIRE images, Glenn *et al.* [116] resolved 60% (250 μm) to 43% (500 μm) of the background to source counts, especially sources below the individual point source detection level in maps. Methods involving stacking analysis resolve more of the background with recent analysis suggesting a resolved fraction greater than 90% [117]. The sources that make up the far-infrared/submillimetre background are dusty SFGs predominantly at high redshifts (*z*>1). They are likely the dominant contribution to the cosmic star-formation rate density during the peak epoch of galaxy formation at *z*∼2–3. We refer the reader to the comprehensive review by Casey *et al.* [5] for properties of these galaxies.

Since COBE/DIRBE and FIRAS CIB intensity measurements, the experimental focus has been on measurements related to the spatial anisotropies and the angular power spectrum of CIB intensity [118–120]. The power spectrum at 60 and 100 μm with IRAS allowed studies related to the spatial distribution properties of Galactic dust and an estimate of the total intensity of extragalactic sources through clustering and Poisson noise at small angular scales (e.g. [37]). At 90 and 170 μm, fluctuation measurements have also been attempted with ISO [121,122]. Significant improvements in our ability to remove Galactic emission and detect extragalactic fluctuations have come from more recent experiments including Spitzer/MIPS at 160 μm [123], AKARI at 90 μm [124], and BLAST at 250, 350 and 500 μm [125]. Currently, the best measurements of CIB power spectrum at a few degree to 10 arcsec angular scales are from Herschel/SPIRE [126–128], while best measurements at larger angular scales and spanning the whole sky are from Planck [129,130]. While there was a mismatch between Herschel/SPIRE and Planck CIB power spectra at overlapping angular scales with first measurements, this difference has mostly gone away with the latest flux calibration of Planck/HFI data. In combination, Planck and Herschel allow studies of the CIB angular power spectrum from large linear scales with Planck to nonlinear 1-halo term [107] with Herschel maps. The measurements are useful to describe the spatial distribution of faint galaxies that make up the CIB and the relationship between far-IR luminosity to dark matter halo mass [128,130–133].

Moving beyond the anisotropy power spectra, CIB fluctuations have also been used for cross-correlation studies. For example, far-IR galaxies are correlated with unresolved near-IR background detected with Spitzer and the cross-correlation of near- and far-IR backgrounds improves models related to the dust distribution within dark matter halos [108]. Sources that make up CIB are mostly at *z*>1. The foreground dark matter potentials that are responsible for lensing of the CMB are mostly at *z*∼1–2 and CIB provides one of the best tracers of the projected dark matter related to CMB lensing [134]. This cross-correlation of CMB lensing with CIB maps has been studied with Planck [135] and for detections of CMB lensing signal in the B-modes of CMB polarization [136,137].

Additional future applications involving the far-IR/submillimetre background include lensing of CIB fluctuations, that is CIB at *z*∼3 is expected to be gravitationally lensed by structures at *z*<1, the search for a diffuse CIB components including intra-halo dust in dark matter halos that extend beyond the dusty discs of SFGs, and a detailed statistical comparison of dust in emission seen with CIB versus dust seen in absorption through extinction studies [138]. While the recent focus has been on CIB fluctuations and their applications, the absolute CIB intensity still remains uncertain at the level of 30% and must be improved down to sub-per cent level if detailed comparisons are to be made on the sources responsible for CIB versus diffuse emission sources at far-IR wavelengths. The proposed Primordial Inflation Explorer (PIXIE) [139] has the capability to achieve such a measurement. Its sensitivity should also be adequate for a detection of the CIB dipole allowing a comparison of the CIB and CMB dipoles.

### 1.6 Microwaves

As shown in figure 1, the CMB, peaking at millimetre wavelengths between sub-millimetre and radio, is the dominant background intensity across all wavelengths in the electromagnetic spectrum. Its total integrated intensity of 960 nW m^−2^ sr^−1^ is roughly a factor of 30 higher than the integrated intensity of the infrared background, which remains the next highest energetic component of the universe. Since its accidental discovery roughly 50 years ago, the high background intensity or photon energy density has facilitated its wide applications in cosmology, especially with spatial anisotropies and polarization using a large number of ground and space-based experiments, including COBE, WMAP and Planck. For recent reviews on CMB theory and experimental data summaries we refer the reader to Durrer [140] and Komatsu *et al.* [141].

While most of the experimental work in CMB concentrates on anisotropies and polarization, the best measurement of CMB spectrum, and correspondingly the best measurement of any EBL component from γ-ray to radio, comes from COBE/FIRAS. It is described by a Planck function with a blackbody temperature of 2.7260±0.0013 [2], with spectral departure from blackbody currently limited by the data to be at the level of *δI*_*ν*_/*I*_*ν*_<10^−5^ [23,24]. Distortions to the spectrum are expected at the micro- and nano-kelvin level (for a general review, see Chluba [142]). Detection and detailed study of these distortions, generated both during the early universe and at late times, remain a primary scientific goal for a next-generation CMB experiment, such as PIXIE [139], with sensitivity at least a factor of 30–100 better than FIRAS.

A well-known cosmological test related to the CMB temperature anisotropy power spectrum involves the location of the first acoustic peak in the multi-polar space [143]. The CMB power spectrum from experiments like Planck now reveal the multiple acoustic peak structure in the anisotropy power spectrum from multipole moments 2 to around 2500 and across at least eight peaks. Along with constraints on cosmological parameters [7], these observations now provide evidence for an initial spectrum of scale-invariant adiabatic density perturbations as expected under models involving inflation. It has been argued for a while that the smoking-gun signature of inflation would be the detection of stochastic background of gravitational waves associated with it. These gravitational waves produce a distinct signature in the polarization of CMB in the form of a contribution to the curl, or magnetic-like, component of the polarization [144]. While polarization from density, or scalar, perturbations dominates, due to the fact they have no handedness, there is no contribution to curl mode polarization from density perturbations. Thus the current generation, and one focus of next generation measurements, involves CMB polarization and especially detailed characterization of the B-modes of polarization.

In transit to us, CMB photons also encounter the large-scale structure that defines the local universe; thus, several aspects of photon properties, such as the frequency or the direction of propagation, are affected. In the reionization epoch, variations are also imprinted when photons are scattered via electrons, moving with respect to the CMB. Though these secondary effects are in some cases insignificant compared to primary fluctuations, they leave certain imprints in the anisotropy structure and induce higher order correlations. A well-studied example of such a secondary effect with current generation CMB experiments is lensing of the CMB [145], with a significant detection of the lensing effect in Planck [146]. The lensing of CMB is useful for cosmological applications involving structure formation and signatures left over by massive neutrinos. A Stage IV CMB experiment will be able to reach the neutrino mass threshold expected given the neutrino oscillation experiments [147]. As discussed with respect to γ-ray background, CMB lensing traces the large-scale structure that is also visible at other wavelengths. Therefore, improvements in our understanding of the nature of dark matter and faint sources at each of the backgrounds will likely come from cross-correlation studies. These are new topics in cosmology that will likely be improved over the coming years.

### 1.7 Radio

The cosmic radio background has been measured at multiple frequencies and in recent years with the balloon-borne ARCADE 2 experiment from 3 to 90 GHz [25]. When compared to CMB, Galactic synchrotron background, and extragalactic point sources, ARCADE 2 measured an excess radio background. For example, at 3 GHz ARCADE measured the equivalent antenna temperature to be 65 mK, once corrected for CMB and Galactic emission. At the same frequency, the known radio galaxy counts contribute about 30 mK. If undetected radio sources account for the excess seen in ARCADE 2 they will need to form an extra peak in the Euclidean-normalized number counts of radio sources at flux densities around 1–100 nJy, but an explanation involving point sources is ruled by various deep radio observations and other arguments (e.g. [148–150]). The excess has also motivated alternative suggestions, such as decaying WIMP dark matter (e.g. [151]). A reanalysis of the Galactic synchrotron emission using multiple components instead of the single slab model for the Galactic plane synchrotron emission used by the ARCADE team, however, suggests that there is likely no excess over the background produced by known sources [152]. Future attempts to improve the radio background will thus likely also involve improvements to understanding and modelling of the Galactic radio foreground.

Current and next-generation experiments will likely focus more on the long wavelength radio background at frequencies around 100 MHz. These experiments are driven by the need to characterize the background intensity spectrum to study the global signature associated with 21 cm spin-flip transition of HI from the epoch of reionization. The global signal involves a strong absorption feature around 60–100 mK, associated with adiabatic cooling of gas, followed by a weak emission during the epoch of reionization [153]. Detection of the expected absorption feature in the background intensity spectrum at frequencies around 60 MHz is challenging due to the large Galactic foreground at these low radio frequencies. Technology development studies are underway to pursue such a measurement from the Moon, including the lunar orbiter DARE (Dark Ages Radio Explorer) [154]. There are also a host of experiments underway at frequencies above 100 MHz focused on the intensity fluctuations, especially the power spectrum of 21 cm background during reionization and for absolute measurements of the sky intensity (see review by Pritchard & Loeb [155]).

## 2. Summary

This review covers the measurements related to the EBL intensity from γ-rays to radio in the electromagnetic spectrum over 20 decades in wavelength. The CMB remains the best measured spectrum with an accuracy better than 1%. The measurements related to the COB, centred at 1 μm, are impacted by the large zodiacal light intensity associated with IPD-scattered sunlight in the inner Solar System. The best measurements of COB come from an indirect technique involving the absorption of γ-ray photons emitted by bright blazars and other active sources in the universe. The CIB at wavelengths centred around 100 μm established an energetically important intensity level comparable to the optical background. This eventually resulted in the discovery of dusty, starbursting galaxies with large aperture telescopes and a deeper understanding of their importance in galaxy formation and evolution. The soft X-ray/EUV extragalactic background at wavelengths of 10–100 nm remains mostly unexplored, but is unlikely to be achieved easily due to the absorption of the extragalactic photons by the intervening neutral intergalactic medium and the interstellar medium of our Galaxy. We also summarize our understanding of spatial anisotropies of these backgrounds and the cosmological/astrophysical applications with angular power spectra of intensity fluctuations across the sky. We motivate a precise direct measurement of the COB between 0.1 and 5 μm using a small aperture telescope observing from either the outer Solar System or out of the ecliptic plane. Other future applications include improving our understanding of the background at TeV energies, improving the MeV background over the previous measurements with COMPTEL, radio background and the spectral distortions to CMB and CIB.

## Data accessibility

Electronic files listing the wavelength and intensity (in units of nW m^−2^ sr^−1^ as plotted in figure 1) and the electronic versions of the figures are available from herschel.uci.edu.
